# Spatiotemporal Analysis of B Cell- and Antibody Secreting Cell-Subsets in Human Melanoma Reveals Metastasis-, Tumor Stage-, and Age-Associated Dynamics

**DOI:** 10.3389/fcell.2021.677944

**Published:** 2021-05-21

**Authors:** Minyi Chen, Franziska Werner, Christine Wagner, Martin Simon, Erika Richtig, Kirsten D. Mertz, Johannes Griss, Stephan N. Wagner

**Affiliations:** ^1^Laboratory of Molecular Dermato-Oncology and Tumor Immunology, Department of Dermatology, Medical University of Vienna, Vienna, Austria; ^2^Department of Dermatology, Medical University of Graz, Graz, Austria; ^3^Cantonal Hospital Baselland, Institute of Pathology, Liestal, Switzerland; ^4^University of Basel, Basel, Switzerland; ^5^Department of Dermatology, Medical University of Vienna, Vienna, Austria

**Keywords:** tumor-associated B cells, memory B cells, plasma cells, human melanoma, tumor microenvironment, multiplex immunohistochemistry, spatiotemporal dynamics

## Abstract

**Background:** The role of tumor-associated B cells in human cancer is only starting to emerge. B cells typically undergo a series of developmental changes in phenotype and function, however, data on the composition of the B cell population in human melanoma are largely absent including changes during tumor progression and their potential clinical significance.

**Methods:** In this study, we compared the number and distribution of six major B cell and antibody secreting cell subpopulations outside tertiary lymphoid structures in whole tumor sections of 154 human cutaneous melanoma samples (53 primary tumors without subsequent metastasis, 44 primary tumors with metastasis, 57 metastatic samples) obtained by seven color multiplex immunohistochemistry and automated tissue imaging and analysis.

**Results:** In primary melanomas, we observed the highest numbers for plasmablast-like, memory-like, and activated B cell subtypes. These cells showed a patchy, predominant paratumoral distribution at the invasive tumor-stroma margin. Plasma cell-like cells were hardly detected, germinal center- and transitional/regulatory-like B cells not at all. Of the major clinicopathologic prognostic factors for primary melanomas, metastasis was associated with decreased memory-like B cell numbers and a higher age associated with higher plasmablast-like cell numbers. When we compared the composition of B cell subpopulations in primary melanomas and metastatic samples, we found a significantly higher proportion of plasma cell-like cells at distant metastatic sites and a higher proportion of memory-like B cells at locoregional than distant metastatic sites. Both cell types were detected mainly in the para- and intratumoral stroma.

**Conclusion:** These data provide a first comprehensive and comparative spatiotemporal analysis of major B cell and antibody secreting cell subpopulations in human melanoma and describe metastasis-, tumor stage-, and age-associated dynamics, an important premise for B cell-related biomarker and therapy studies.

## Introduction

The tumor immune microenvironment critically regulates tumor initiation, progression and response to therapy (reviewed in Binnewies et al., 2018). Though B cells constitute a significant part of this microenvironment, the exploration of their role in human cancer has just begun (reviewed in Fridman et al., 2021).

Syngeneic mouse cancer models have shown that tumor-associated B cells (TAB) and antibody secreting cells (ASC), such as plasmablasts, can promote tumor progression (de Visser et al., 2005; Ammirante et al., 2010) and inhibit (Affara et al., 2014; Shalapour et al., 2015, 2017; Gunderson et al., 2016) but also support anti-tumor T cell-dependent therapy responses (Lu et al., 2020). In melanoma, syngeneic mouse models revealed both pro- as well as anti-tumorigenic effects of B cells (reviewed in Fremd et al., 2013), while advanced models such as genetically engineered mouse models or xenotransplantation models suffer from inadequate tumor infiltration by B cells or other immune cells of the tumor microenvironment (Hooijkaas et al., 2012). These data underline the importance of studies in human tissue samples.

In human melanoma, phenotypic analysis showed that the tumor microenvironment contains CD20^+^ TAB (reviewed in Ladányi, 2015) and CD138^+^ or IgA^+^CD138^+^ ASC, which are primarily found at the invasive tumor-stroma margin (Erdag et al., 2012; Bosisio et al., 2016; Griss et al., 2019). However, existing data on their impact on disease progression and outcome are inconsistent. Initial studies on the association of TAB numbers in primary human melanomas with patient survival employed CD20-immunostaining and reported on higher numbers in histological subtypes with a worse prognosis (Hillen et al., 2008) or on the association of higher percentages of CD20^+^ TAB within tumor-infiltrating lymphocytes with a worse patient prognosis (Martinez-Rodriguez et al., 2014). These data were supported by observations from melanoma metastases where a 7-marker protein signature, including CD20, negatively predicted overall and recurrence-free patient survival (Meyer et al., 2012). Also in other human cancer types, including ductal carcinoma *in situ* of the breast, pancreatic ductal carcinoma and non-small cell lung, colorectal, oral hypopharynx, prostate and metastatic ovarian cancers, reports do exist that correlate infiltration with CD20^+^ or CD19^+^ TAB with poor patient disease outcome, tumor recurrence and/or progression (reviewed in Mohammed et al., 2013; Woo et al., 2014; Dong et al., 2006; Wouters and Nelson, 2018).

These studies, however, are contrasted by several other independent reports showing higher numbers or densities of CD20^+^ TAB to be associated with improved patient survival, such as in melanoma metastases (Erdag et al., 2012) and primary cutaneous melanomas (Ladányi et al., 2011; Garg et al., 2016). These data are in line with reports from other human cancer types, including (triple-negative) inflammatory breast, epithelial and high-grade serous ovarian, non-small cell lung, early cervical squamous cell, metastatic colorectal, gastric, hepatocellular, pancreatic ductal, esophageal, biliary tract, muscle invasive bladder, oral hypopharynx, tongue squamous cell cancers, soft tissue sarcoma and mesothelioma, where high intratumoral CD20^+^ or CD19^+^ TAB numbers or densities alone or sometimes together with CD3^+^ or CD8^+^ T cells are associated with a favorable disease outcome (Linnebacher and Maletzki, 2012; Bindea et al., 2013; Lao et al., 2016; Schwartz et al., 2016; Wouters and Nelson, 2018; Helmink et al., 2020; Fridman et al., 2021).

In studies including additional expression of CD138, a marker associated with plasma cell differentiation, high CD20 together with CD138 expression correlated with a higher tumor grade in epithelial ovarian cancer and immune cell-associated CD138 expression alone with poorer overall and cancer-specific patient survival (Lundgren et al., 2016). Similarly, infiltration of CD20^+^ TAB with CD138 expression into primary operable ductal invasive breast cancer was associated with poorer cancer-specific survival (Mohammed et al., 2013) and plasma cell enrichment in G1/2 papillary/acinar adenocarcinomas described as an independent negative prognostic factor (Kurebayashi et al., 2016). In contrast, a favorable prognostic effect was described for CD138^+^ cell infiltration in colorectal, esophageal and gastric cancers (reviewed in Wouters and Nelson, 2018; Fridman et al., 2021). In human melanoma metastases, increased CD138^+^ plasma cell counts showed a trend to better patient survival (Erdag et al., 2012) and patients with primary melanomas of > 2 mm in thickness and enriched for sheets/clusters of CD138^+^ IgA-expressing plasma cells had a worse overall survival. In contrast, plasma cell-sparse melanomas had a significantly better survival than plasma cell-rich tumors and a trend toward a better survival than plasma cell-negative tumors (Bosisio et al., 2016).

Together, these data clearly pinpoint the presence of TAB and ASC in different human cancers, cancer subtypes and tumor stages, but indicate that these B cells may play varied roles. An attractive hypothesis for the varied roles of B cells is the presence of different TAB and ASC subpopulations. However, such data are lacking, mainly because of the limited ability to apply complex marker combinations required to identify such B cell phenotypes. We have recently shown that B cells from human melanoma are not only essential to sustain inflammation and CD8^+^ T cell numbers in the tumor microenvironment but also can directly augment T cell activation by immune checkpoint blockade (Griss et al., 2019). We were also the first to report increased B cell numbers to predict improved response and survival of melanoma patients receiving immune checkpoint blockade. These data were most recently supported and extended by independent analyses of melanoma tertiary lymphoid structures (TLS), where tumor-associated B cells are thought to be educated (Cabrita et al., 2020; Helmink et al., 2020). Together, these and our studies describe B cells to play an unexpected essential role in T cell-based anti-tumor immunity and the responsible B cell phenotypes to typically having undergone antigen-dependent activation and class switch recombination (Griss et al., 2019; Helmink et al., 2020). Despite their presumably high relevance for anti-tumor immune responses and immunotherapeutic strategies, data on the presence of these B cell subpopulations and their distribution in human melanoma are mostly missing so far.

Here we present a systematic comparative spatiotemporal analysis for six different antigen-experienced B cell and antibody secreting cell subpopulations in a series of 154 human melanoma samples. Using seven color multiplex immunohistochemistry and automated tissue imaging and analysis of whole tumor sections (Griss et al., 2019), we detected metastasis-, tumor stage-, and age-associated dynamics in the composition of these subpopulations.

## Materials and Methods

### Patient Cohorts

Whole tissue sections were obtained from cutaneous primary melanomas of caucasian patients who underwent surgery between 2002 and 2016 at the Cantonal Hospital Baselland, Liestal, Switzerland, and between 2004 and 2020 at the Department of Dermatology, Medical University of Graz, Austria. Tumor samples from Graz were provided by the Biobank Graz of the Medical University. All tumors were obtained with informed patients’ consent and the pathology files retrieved as approved by the local Ethics Committees (EKNZ vote BASEC 2016-01499 for Liestal; 32-238 ex 19/20 for Graz).

Histological diagnoses were made by board-certified pathologists from the Cantonal Hospital Baselland, Liestal, and board-certified dermatologists at the Department of Dermatology, Medical University of Graz, in some cases together with external board-certified pathologists. Diagnoses were reviewed by two authors of this study, a board-certified pathologist (KM) and a board-certified dermatologist (SW). The respective clinicopathologic information was recorded in Tables 1, 2. As desmoplastic melanomas show a distinct clinical behavior, they were not included into this study (Lens et al., 2005).

**TABLE 1:** Clinical and histopathological summary of melanoma patients with primary tumors without subsequent metastasis.

**No. of patients**		**53**
Follow-up (months)	Mean	84
	Median	98
	Range	8–194
Age	Mean	64
	Median	68
	Range	31–93
Breslow depth (thickness in mm)	Mean	2.49
	Median	1.75
	Range	0.36–10
Location	Extremities	20
	Head/neck	3
	Trunk	30
Ulceration	Present	23
	Absent	30
Histotype*	SSM	40
	NM	8
	ALM	4
	NOS	1
Sex	Male	33
	Female	20

*SSM, superficial spreading melanoma; NM, nodular melanoma; ALM, acral lentiginous melanoma; NOS, not otherwise specified.

**TABLE 2:** Clinical and histopathological summary of melanoma patients with primary tumors with subsequent metastasis.

**No. of patients**		**44**
Follow-up (months)*	Mean	54
	Median	32
	Range	0–231
Age	Mean	65
	Median	68
	Range	19–91
Breslow depth (thickness in mm)**	Mean	4.71
	Median	2.65
	Range	0.7–17
Location**	Extremities	15
	Head/neck	7
	Trunk	21
Ulceration**	Present	24
	Absent	19
Histotype***	SSM	20
	NM	19
	ALM	2
	LMM	1
	NOS	2
Sex	Male	30
	Female	14

*Seven samples without follow-up information (after metastasis). **Five samples without exact information about Breslow depth, one about location, one about ulceration. ***SSM, superficial spreading melanoma; NM, nodular melanoma; ALM, acral lentiginous melanoma; LMM, lentigo maligna melanoma; NOS, not otherwise specified.

The cohort included 97 patients with primary cutaneous melanoma, aged between 19 and 93 years at the time of first diagnosis. 53 patients presented without metastasis within a follow-up of a maximum 194 months interval (mean: 84 months, Table 1). 44 patients developed metastasis within a follow-up of a maximum 231 months interval (mean: 54 months, Table 2). From 10 of the latter patients, additional 16 metastatic samples were collected at the time of first diagnosis. These early metastatic samples almost exclusively consisted of locoregional skin and clinically detectable (macroscopic) nodal metastases, where tumor deposits had completely replaced lymph node tissue or could be histologically clearly separated from remains of the lymphatic tissue.

None of these patients received local or systemic antitumor treatment before surgery of the primary tumor.

### Seven Color Multiplex Immunohistochemical Staining for TAB and ASC Subpopulations

Tumor tissue analysis and read-out were approved by the Ethics Committee of the Medical University of Vienna (ethics vote 1999/2019). Four micrometer sections from formalin-fixed paraffin-embedded blocks were used. Staining parameters for each primary antibody were optimized using human tonsil tissue and representative study samples. The complete multiplex immunostaining procedure was designed as previously described (Carstens et al., 2017; Gorris et al., 2018; Griss et al., 2019). Antibodies used in stainings were against: CD19 (1:250 dilution, Abcam, clone EPR5906, catalogue number #134114), CD20 (1:2000, Agilent, clone L26, M0755), CD38 (1:450, Agilent, clone AT13/5, M7077), CD138 (1:450, Agilent, clone MI15, M7228), CD27 (1:500, Abcam, clone EPR8569, #ab 131254), CD5 (1:500, Novocastra, clone 4C7, CD5-4C7-L-CE).

Tissue sections were subjected to six rounds of immunohistochemical staining after dewaxing. Each round of staining started with a 30 min heat-induced antigen retrieval step with either citrate buffer (pH 6.0) or Tris-EDTA buffer (pH 9.0), respectively, a subsequent 30 min fixation step with neutral 7.5% formaldehyde (SAV Liquid Production) and a 15 min blocking step using 20% normal goat serum (Agilent, X0907), followed by successive incubations with primary antibody, biotinylated anti-mouse or -rabbit secondary antibody, Streptavidin-HRP (Dako, K5003) and Opal fluorophore dye (Akoya Biosciences). Each antibody was assigned to one of the fluorophores Opal 520, Opal 540, Opal 570, Opal 620, Opal 650, and Opal 690 (Akoya; FP1487001KT, FP1494001KT, FP1488001KT, FP1495001KT, FP1496001KT and FP1497001KT) diluted in 1X Plus Amplification Diluent (Akoya, FP1498). After six rounds of antibody stainings, nuclei were counterstained with DAPI (Akoya, FP1490) and slides mounted with PermaFluor fluorescence mounting medium (Thermo Fisher Scientific, TA-030-FM). Stainings with single primary antibodies were run in parallel to control for false positive (incomplete stripping of antibody-tyramide complexes) and false negative results (antigen masking by multiple antibodies, “umbrella-effect”) as well as for spillover effects (detection of fluorophores in adjacent channels) as described by us before (Griss et al., 2019). Reproducibility was controlled by a reference slide in each run, antibody batches were not changed in this study. Negative controls included concentration-matched isotype stainings and stainings without primary antibodies (Kaufman et al., 2013). To detect CD19^*low*^ plasma cell-like ASC, the concentration of the primary anti-CD19 antibody was adapted to allowing for detection of cellular patterns and frequencies obtained by CD138^+^ pooled IgA/IgG^+^ stainings on human tonsil and melanoma (Griss et al., 2019).

### Automated Acquisition and Quantification of TAB and ASC Subpopulations

Multiplexed slides of the whole tumor sections were scanned on a Vectra 3 Automated Quantitative Pathology Imaging System (version 3.0.5., Akoya) and, after spectral unmixing, analyzed with inForm^®^ Tissue Finder^TM^ (version 2.4.1, Akoya) as described (Carstens et al., 2017; Gorris et al., 2018; Griss et al., 2019). An unstained representative tumor section was used to determine autofluorescence. Tumor areas with closely packed clusters of lymphoid cells (e.g., TLS) did not allow for an unambiguous allocation of fluorophore signals to a single cell and were excluded from our analysis as were tumor areas of ulceration. If any, remaining lymphatic tissue in early locoregional nodal metastases was also excluded.

**FIGURE 1:**
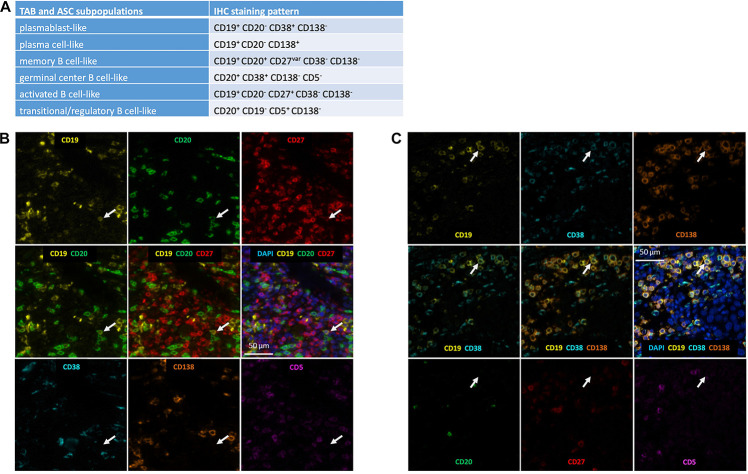
Detection of TAB and ASC subpopulations in human melanoma. **(A)** Marker combinations used to identify TAB and ASC subpopulations by seven color multiplex immunostaining. Identification of **(B)** a CD19^+^CD20^+^CD27^+^ memory-like TAB and **(C)** a CD19^+^CD38^+^CD138^+^ plasma cell-like ASC. Serial images for the different markers: positive markers are given in the upper row; composite images of positive markers are given in the middle row, together with DAPI nuclear staining in the middle right; negative markers are given in the lower row. Arrows depict the same cell being representative for the respective TAB and ASC subpopulation. Scale bars represent 50 μm.

We identified TAB and ASC subpopulations in whole tumor sections by differential CD19, CD20, CD38, CD138, CD27 and CD5 expression as described by us before (Griss et al., 2019): (i) CD19^+^ CD20^–^ CD38^+^ CD138^–^ as plasmablast-like, (ii) CD19^+^ CD20^–^ CD138^+^ as plasma cell-like, (iii) CD19^+^ CD20^+^ CD38^–^ CD138^–^ CD27^*var*^ as memory-like B cells, (iv) CD20^+^ CD38^+^ CD138^–^ CD5^–^ as germinal center-like B cells, (v) CD19^+^ CD20^–^ CD38^–^ CD138^–^ CD27^+^ as activated B cells, (vi) CD20^+^ CD19^–^ CD138^–^ CD5^+^ as transitional/regulatory-like B cells and (vii) other cells (Figure 1). The CD19 staining was optimized for detection of CD19^*low*^ plasma cell-like cells (see above) and these cells could be detected at significant numbers, still they may be slightly underrepresented. As expression of CD27 can be downregulated on tumor-infiltrating B cells (Nielsen et al., 2012; Hegde et al., 2016), also activated B cells may be underrepresented. All phenotyping and subsequent quantifications were performed blinded to the sample identity.

### Statistical Analysis

Cell-level data (i.e., intensities per channel for the cell compartments “membrane,” “cytoplasm” and “nucleus”) were exported from inForm^®^ Tissue Finder^TM^ as text files and processed using R (version 4.0.3). Intensity thresholds were manually adjusted after manual inspection of every slide. Based on these thresholds, markers were defined as positive or negative and cells assigned to specific phenotypes based on the above marker combinations.

## Results

### Experimental Strategy

TAB in primary human melanomas show a rather patchy and inhomogeneous infiltration pattern with sometimes predominant paratumoral, intratumoral or mixed distribution (Garg et al., 2016). We therefore decided to analyze whole tissue sections of 154 human cutaneous melanoma samples from four different cohorts representing different stages of melanoma progression by seven color multiplex immunohistochemistry and automated tissue imaging and analysis for the presence and distribution of six different B cell subpopulations (activated, memory-like, germinal center-like and transitional/regulatory-like TAB as well as plasmablast-like and plasma cell-like ASC) outside TLS.

We first determined the absolute frequencies of each TAB and ASC subpopulations in tumor samples from 97 primary human melanomas and their association with the most important categorical clinicopathologic parameters.

For an association with disease progression, we compared the composition of the TAB/ASC population in primary tumor samples to that in 16 early locoregional and 41 late distant melanoma metastases. Data for the composition of the TAB/ASC population at distant metastatic samples were taken from own published data that have been collected with exactly the same staining, imaging and analysis approach in melanoma skin metastases (Griss et al., 2019).

### Frequencies of Distinct B Cell and Antibody Secreting Cell Subpopulations in Primary Melanomas Are Associated With Prognostic Clinicopathologic Parameters

In line with previous reports on CD20^+^ TAB (Garg et al., 2016), TAB and ASC subpopulations showed a rather patchy, predominant paratumoral distribution at the invasive tumor-stroma margin and sometimes an intratumoral as well as a mixed intratumoral and paratumoral infiltration pattern. We therefore decided to use whole tumor sections to stain 97 cutaneous primary human melanomas samples for the presence of six different B cell subpopulations outside TLS. We found TAB and ASC subpopulations in 65 of 97 primary melanoma samples (67%, Table 3). These subpopulations could be classified as activated and memory-like TAB as well as plasmablast-like and plasma cell-like ASC (Figure 1). Germinal center-like and transitional/regulatory-like TAB were not detected. Frequencies were highest for plasmablast-like ASC, followed by memory-like and activated TAB subpopulations. Plasma cell-like ASC were detected only at very low numbers (Table 3).

**TABLE 3:** Summary of multiplex immunohistochemistry staining results in primary melanoma samples.

	**Primary melanomas without metastasis**	**Primary melanomas with metastasis**
No. of samples	53	44
No. of samples with TAB and/or ASC subpopulations	41	24
No. of cells/mm^2^ tumor area		
Activated TAB, range:	0–56	0–38
Mean:	4.0 ± 9.3	2.2 ± 6.3
Memory-like TAB*, range:	0–73	0–49
Mean:	5.9 ± 14.5	2.7 ± 9.5
Plasmablast-like ASC, range:	0–103	0–29
Mean:	7.7 ± 18.3	2.5 ± 6.5
Plasma cell-like ASC, range:	0–5	0–7
Mean:	0.6 ± 1.1	0.4 ± 1.1

*p ≤ 0.05 between primary melanomas without vs. with metastasis.

**FIGURE 2:**
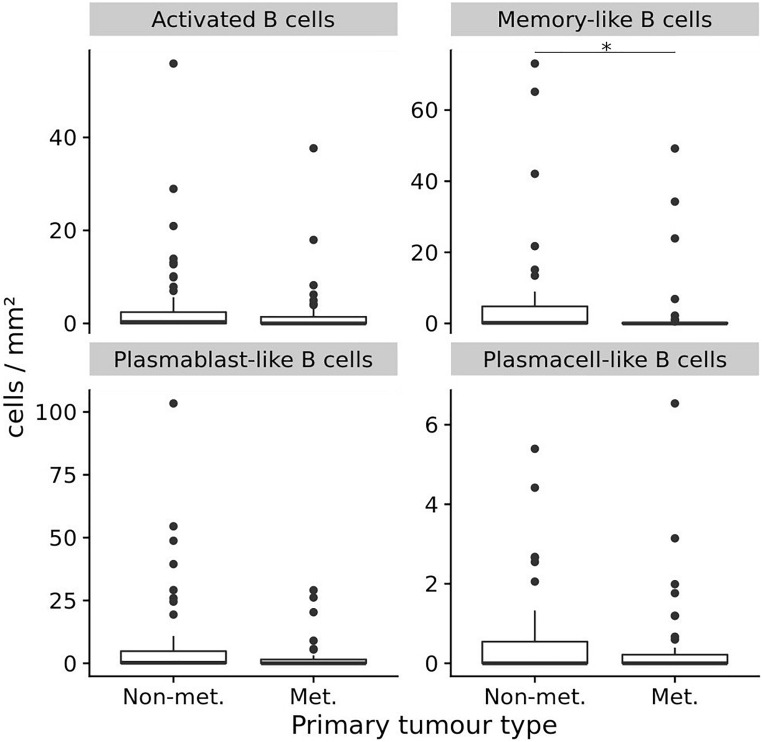
The frequencies (cells/mm^2^) of four different TAB and ASC subpopulations in primary human melanomas and their association with metastasis. Box plots comparing primary tumors that did not metastasize (Non-met.) within a mean follow-up of 84 months vs. those that metastasized (Met.) with a mean follow-up of 54 months. FDRs are 0.02 for memory-like TAB and 0.17 for plasmablast-like ASC. In boxplots lower and upper hinges correspond to first and third quartiles and center lines to medians. Upper whiskers extend to the largest value within 1.5 times the interquartile range. Outliers are shown as black circles. ^∗^*p* ≤ 0.05.

We then compared primary melanoma samples for metastasis, the single most important prognostic factor for melanoma patients, and observed that primary melanomas with metastasis contained a lower number of TAB and ASC containing tumors than primary melanomas without metastasis (Table 3 and Figure 2). Primary tumors with subsequent metastasis contained significantly less memory-like TAB (mean 5.9 ± 14.5 vs. 2.7 ± 9.5 cells/mm^2^, *p* = 0.02, CI 95% = –0.75 to 0, Bonferroni corrected Wilcoxon Rank Sum test) and a trend toward a decreased frequency of plasmablast-like cells (mean 7.7 ± 18.3 vs. 2.5 ± 6.5, *p* = 0.17, CI 95% = –0.41 to 0, Bonferroni corrected Wilcoxon Rank Sum test) compared to tumor samples without metastasis (Table 3 and Figure 2).

**FIGURE 3:**
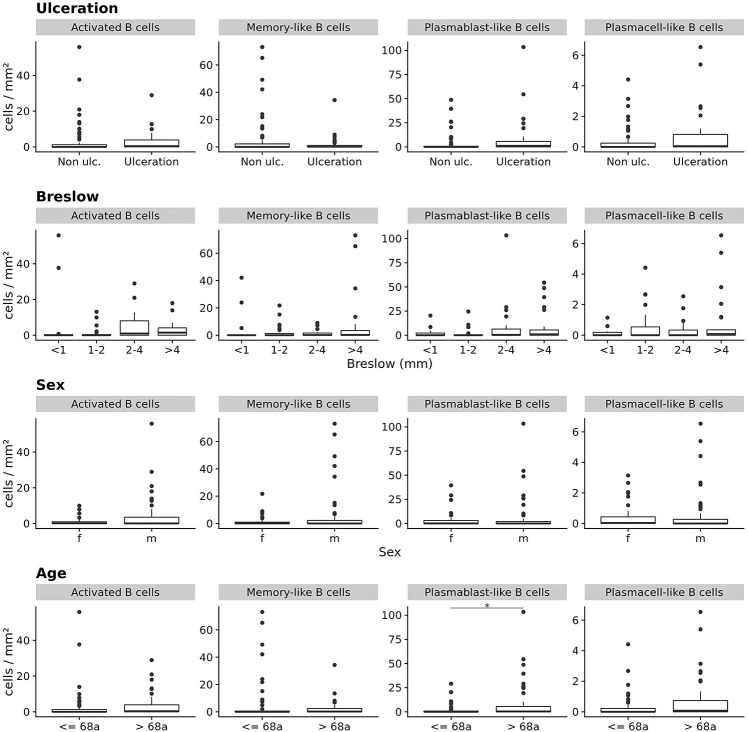
The frequencies (cells/mm^2^) of four different TAB and ASC subpopulations in primary human melanomas and their association with categorial prognostic clinicopathologic parameters. Box plots comparing primary tumors with and without ulceration, with increasing Breslow depths, for sex and age (from top to bottom). FDR is 0.05 for plasmablast-like ASC and age (median = 68 years). In boxplots lower and upper hinges correspond to first and third quartiles and center lines to medians. Upper whiskers extend to the largest value within 1.5 times the interquartile range. Outliers are shown as black circles. *p ≤ 0.05.

When we stratified primary melanoma samples for other prognostically important categorical clinicopathologic parameters, we found primary tumor samples from patients with higher age (above the median of 68 years) to contain significantly higher frequencies of plasmablast-like ASC (mean 9.4 ± 20 vs. 2.0 ± 5.3 cells/mm^2^; *p* = 0.05, CI 95% = 0 to 1.3, Bonferroni corrected Wilcoxon Rank Sum test), but not for the other three TAB and ASC subpopulations (Figure 3). Differences for Breslow depth, ulceration and sex were not found (Figure 3).

The increased frequency of plasmablast-like ASC in primary melanomas of patients with higher age was somewhat surprising in view of the reported decrease of mature B cell numbers with age (reviewed in Crooke et al., 2019). We therefore screened each individual tumor sample for the frequency of plasmablast-like ASC and found a small subgroup of primary melanomas with considerably high frequencies. When we then compared the top 10% primary melanomas with highest frequencies of plasmablast-like ASC (*n* = 10) vs. the rest of tumor samples, we found this subgroup significantly driven by higher age and—to a minor degree—by higher Breslow depth (*p* < 0.01 and *p* = 0.09, respectively, ANCOVA), but not by sex or the presence of ulceration.

Thus, a decreased frequency of memory-like TAB is associated with metastasis of primary melanomas and an increased frequency of plasmablast-like cells with higher age.

### Frequencies of Distinct B Cell and Antibody Secreting Cell Subpopulations Are Associated With Different Stages of Melanoma Disease

We next hypothesized that the observed changes in the frequency of TAB and ASC subpopulations with metastasis may also give a hint on changes in the frequency or composition of TAB and ASC subpopulations in further stages of melanoma progression. We thus compared primary tumors with locoregional and distant metastatic tumor sites.

Therefore, an additional 16 early locoregional metastases were stained by multiplex immunohistochemistry for TAB and ASC subpopulations. In nodal tumor samples, tumor deposits had completely replaced lymph node tissue or could be histologically clearly separated from the remaining lymphatic tissue. In the rare cases where some lymphatic tissue was left, we also observed infiltration of the subcapsular region. In tumor samples from early locoregional metastases, TAB and ASC subpopulations were detected primarily in stromal septa within the tumor and paratumorally at the invasive tumor-stroma margin, a pattern comparable to that reported previously for distant metastatic sites (Griss et al., 2019).

**FIGURE 4:**
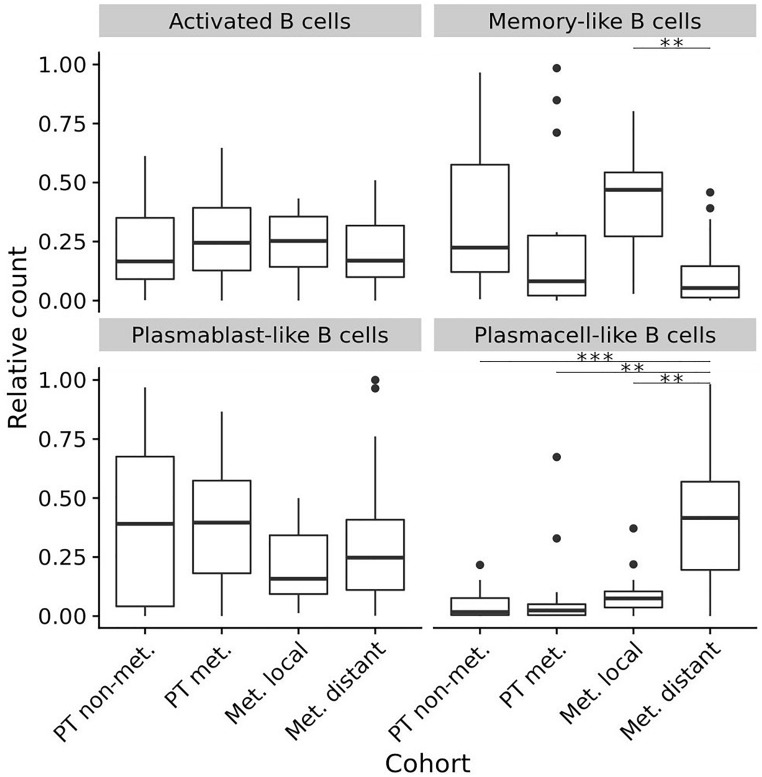
Composition (relative frequencies) of four different TAB and ASC subpopulations in different stages of human melanoma disease. Box plots comparing primary tumors that did not metastasize (PT non-met., *n* = 22) with primary tumors that metastasized (PT met., *n* = 14), early locoregional metastatic sites (Met. local, *n* = 15) and late distant metastatic sites (Met. distant, *n* = 33). FDRs (adjusted) for memory-like TAB at early locoregional metastatic sites are 0.08 and 0.001 compared to primary tumors that metastasized and late distant metastatic sites, respectively. FDRs (adjusted) for plasma cell-like ASC at late distant metastatic sites are < 0.001 compared to primary tumors that did not metastasize, and 0.001 compared to both primary tumors that metastasized as well as early locoregional metastatic sites. In boxplots lower and upper hinges correspond to first and third quartiles and center lines to medians. Upper whiskers extend to the largest value within 1.5 times the interquartile range. Outliers are shown as black circles. ^∗∗^*p* < 0.01, ^∗∗∗^*p* < 0.001.

To compare these data from primary tumors and early locoregional metastatic sites with those from late distant metastatic sites, we used our own published data that had been generated in distant melanoma metastases by exactly the same staining, imaging and analysis approach (Griss et al., 2019). This data set provides relative frequencies of exactly the same TAB and ASC subpopulations and we compared these to the relative frequencies obtained in the present study. To allow analysis for the several TAB and ASC subpopulations within individual tumor samples, we included into this comparison tumor samples with only ≥50 B cell counts as determined by CD20- and/or CD19-immunoreactivity (for sample numbers see legend Figure 4).

While the relative counts for activated TAB did not change between primary tumors, locoregional and distant metastatic sites, we observed significant changes for memory-like TAB and plasma cell-like ASC (Bonferroni corrected *p* < 0.01 for both, Kruskal-Wallis Test). Memory-like TAB at locoregional metastatic sites showed comparable relative counts to primary tumors that did not metastasize but were increased compared to primary tumors with subsequent metastasis and distant metastatic sites (Bonferroni corrected *p* = 0.08 and *p* < 0.001, CI 95% –0.46 to –0.02 and 0.19 to 0.46, respectively, Wilcoxon rank sum test) (Figure 4). Plasma cell-like ASC exhibited highest relative counts at distant metastatic sites, these counts were significantly higher than in primary tumors and locoregional metastases (Bonferroni corrected *p* < 0.01 for both, CI 95% –0.47 to –0.17, –0.4 to –0.14, respectively, Wilcoxon rank sum test, Figure 4).

Thus, during disease progression memory-like TAB show highest frequencies at early locoregional metastatic sites whereas plasma cell-like ASC preferentially accumulate at late distant metastatic sites.

## Discussion

TAB and ASC occur in many human cancers but may play varied functional roles dependent on cancer type, genetic and histological subtypes and tumor stages. There is now substantial data that B cells may play an essential role in T cell-based anti-tumor immunity in human melanoma (Griss et al., 2019; Cabrita et al., 2020) by sustaining inflammation and CD8^+^ T cell numbers in the tumor microenvironment and directly augmenting T cell activation by immune checkpoint blockade (Griss et al., 2019). While different TAB and ASC subtypes have been suggested to exert these immunostimulatory functions, reported candidate subtypes are typically antigen-activated and somatically recombined (Griss et al., 2019; Helmink et al., 2020). Beside activated TAB, such subtypes include germinal-center and memory B cells as well as plasmablasts and plasma cells and we have now performed a systematic analysis for the presence of these TAB and ASC subtypes in human melanoma samples comprising different stages of tumor progression. Using seven color multiplex immunohistochemistry and an automated tissue imaging and analysis approach, we identified (i) in primary melanomas highest numbers for plasmablast-like ASC, followed by memory-like and activated TAB, whereas plasma cell-like ASC were detected at comparably very low numbers; (ii) high frequencies of plasmablast-like ASC in primary melanomas to be driven by higher age and, to a lesser extent, by Breslow depth; (iii) an association of metastasis of primary melanomas with decreased counts of memory-like TAB; and (iv) increased relative frequencies for memory-like TAB at locoregional metastatic sites and a preferential enrichment for plasma cell-like ASC at distant metastatic sites.

Activated and memory-like TAB as well as plasmablast- and plasma cell-like ASC have been described by us before in distant human metastatic lesions and this study now confirms their presence also in regional metastatic and in primary melanoma sites. Similar to their distribution in distant metastases, TAB and ASC subpopulations in regional metastases were present in stromal septa within the tumor and at the invasive tumor stroma margin. In primary tumors, TAB and ASC subpopulations showed a rather patchy, predominant paratumoral distribution at the invasive tumor-stroma margin and sometimes an intratumoral as well as a mixed intratumoral and paratumoral infiltration pattern. These patterns are comparable to those reported previously for CD20^+^ B cells in primary melanomas and other cancer types (Bindea et al., 2013; Garg et al., 2016). Also similar to CD20^+^ TAB, the frequencies of distinct TAB and ASC subpopulations in primary human melanomas did not correlate with tumor thickness and ulceration, but with decreased metastasis (Garg et al., 2016). Thus our study further supports the meanwhile prevailing view of an anti-tumorigenic role of B cells in primary human melanoma (reviewed in Fridman et al., 2021) and provides the first evidence for diminution of a distinct TAB subpopulation, namely memory-like B cells in primary tumors with documented metastasis. Memory B cells are critical to the induction of adaptive B cell responses not only to foreign but also to tumor antigens (Seifert and Küppers, 2016). A recent report has highlighted their potential therapeutic role in metastatic human melanoma where memory B cells have been associated with TLS (Helmink et al., 2020) and, together with other antigen-experienced and somatically recombined TAB and ASC subpopulations, linked to response of metastatic disease to immune checkpoint blockade (Griss et al., 2019; Cabrita et al., 2020; Helmink et al., 2020). The demonstrated detection of this B cell subpopulation particularly in TLS of metastatic locoregional nodal sites (Helmink et al., 2020) is also in line with our observation of increased memory-like TAB counts at locoregional sites, which consisted mainly of lymph nodes in our study cohort.

Plasmablast-like cells showed the highest frequency of TAB and ASC subpopulations in primary melanomas. These frequencies, however, were mainly driven by a subgroup of melanoma patients with increased age and Breslow depth. Human B cell populations are known to change quantitatively and qualitatively with increasing age and these changes have a clear impact on anti-tumor immune cell functions, best documented by the increased cancer susceptibility of older adults. In contrast to switched memory B cells, naive B cells and unswitched mature B cells in peripheral blood significantly decrease with age, a phenomenon that is linked to intrinsic mechanisms like decreased mRNA stability of transcription factor E47, which results in decreased induction of activation-induced cytidine deaminase and impaired ability to undergo class switch recombination and antibody secretion (Frasca et al., 2011). These intrinsic mechanisms are complemented by additional extrinsic factors such as defects in T cell help to B cells or increased numbers of regulatory T cells in the peripheral blood (Wagner et al., 2018) as well as structural and functional changes in secondary lymphoid organs of the elderly (reviewed in Crooke et al., 2019). All these intrinsic and extrinsic factors argue against B cell maturation within germinal centers of secondary or tertiary lymphoid structures as a mechanism underlying the increased frequencies of plasmablast-like ASC in a subgroup of primary melanomas. In line with this assumption, we could hardly detect any mature TLS in hematoxylin & eosin or multiplex immunohistochemical stains in this subgroup (own unpublished data). An alternative source for plasmablasts in primary melanomas could be the B1 cell population which can differentiate into short lived IgM + plasmablasts outside germinal centers. Though B1 cells also undergo age-associated changes such as a decrease of numbers in peripheral blood, sub-analyses have shown that with advancing age spontaneous antibody secretion by B1 cells is modified, but not necessarily reduced in terms of the number of IgG-secreting B1 cells and the amount of IgM secretion per cell (Rodriguez-Zhurbenko et al., 2019). B1 cells express substantial levels of the transcription factor BLIMP1, which decrease with age, and of the transcription factor PAX5, which increase with age (Rodriguez-Zhurbenko et al., 2019). As high BLIMP1 levels are required particularly for plasma cell differentiation, one is tempting to speculate that the reduced BLIMP1 levels at higher age could still be sufficient to induce plasmablasts which need significantly less BLIMP1 for differentiation (Nutt et al., 2015). Consistently, the subgroup of primary melanomas with highest numbers of plasmablast-like ASC did not contain somehow comparable numbers of plasma cell-like ASC. Furthermore, recent mouse data indicate that Pax5 downregulation is not required for plasmablast development but is rather essential for accumulation and optimal IgG secretion of long-lived plasma cells with progressing age (Crooke et al., 2019; Liu et al., 2020).

CD138^+^ IgA^+^ plasma cells have been described in human primary melanomas particularly of >2 mm in thickness, where plasma cell-rich tumors—as identified by staining for CD138—had a worse overall survival than plasma-sparse ones (Bosisio et al., 2016). Consistent with our data, both CD138^+^ and CD138^+^ IgA^+^ plasma cells were detected in only a small number of primary melanomas from two independent cohorts (Bosisio et al., 2016). While we could not address an association with overall survival in our study, numbers of plasma cell-like ASC in our study could be associated neither with metastasis nor with prognostic markers such as Breslow depth or the presence of ulceration as reported for CD138^+^ plasma cells (Bosisio et al., 2016). This may be due to the smaller sample size in our study, particularly of tumors with >2mm in thickness, and/or due to different staining and tissue evaluation approaches. These include the usage of multiple markers vs. a single marker for detection of plasma cells. While staining for CD138 alone, which is not exclusively expressed on plasma cells, may have led to overestimation of cell numbers, we cannot exclude a slight underestimation in our study, though we have optimized our CD19 immunostaining approach for detection of CD19^*low*^ plasma cell-like cells. Additional differences are the evaluation of whole tissue samples vs. selected tissue areas and the use of a quantitative automated read-out vs. application of a semiquantitative visual scoring system. Interestingly, the rather low number of plasma cell-like ASC at primary melanoma sites was paralleled by a low number in locoregional metastatic sites but contrasted by a significant enrichment at distant metastatic sites, which comprised skin metastases in our study cohort. An attractive explanation for this observation may provide the reported neogenesis of TLS particularly in human melanoma skin metastases (Cipponi et al., 2012) together with the recent reports on an association of mature TLS (Cabrita et al., 2020; Helmink et al., 2020; Petitprez et al., 2020) with clonal B cell expansion, increased B cell receptor diversity and higher numbers of plasmablasts/plasma cells (Cabrita et al., 2020; Helmink et al., 2020). While some earlier reports describe the presence of TLS-associated cell types such as high endothelial venules (Dieu-Nosjean et al., 2008) and LAMP3^+^ mature dendritic cells (Ladányi et al., 2007) in primary human melanomas, a systematic analysis of numbers, areas, localization and maturation states of TLS at different tumor stages is still lacking.

## Conclusion

Together, this study significantly widens the knowledge of the spectrum of B cell subpopulations and their spatiotemporal dynamics in human melanoma. Our data show a rather inhomogeneous distribution along different stages of human melanoma progression with numbers of memory-like TAB which decrease in primary melanomas that metastasize, but increase at locoregional metastatic sites, and with numbers of plasma cell-like ASC which increase at distant metastatic sites. These variations may have important implications for the biology of human melanoma as well as for the development of B cell-related biomarker and therapy studies.

## Data Availability Statement

Datasets generated for this study are available to any qualified researcher on request to the corresponding author, without undue reservation.

## Ethics Statement

The patients/participants provided their written informed consent to tumor sample collection. Tumor sample collection and the studies involving these tumor samples were reviewed and approved by the Ethikkommission Nordwest- und Zentralschweiz (vote BASEC 2016-01499) and the Ethikkommission der Medizinischen Universität Graz (32-238 ex 1103 19/20). Tumor tissue analysis and read-out was additionally approved by the Ethics Committee of the Medical University of Vienna (ethics vote 1999/2019).

## Author Contributions

JG and SW: conception and design. ER, KM, MC, MS, and SW: clinical data collection and assembly, patient materials. MC, FW, CW, MS, and SW: multiplex immunostaining, automated imaging acquisition, and data read out. JG: statistics. All authors contributed to the manuscript writing and final approval of the manuscript.

## Conflict of Interest

The authors declare that the research was conducted in the absence of any commercial or financial relationships that could be construed as a potential conflict of interest.
